# Charting the future of cardiorenal medicine: a vision for integration, innovation, and impact

**DOI:** 10.3389/fneph.2025.1719673

**Published:** 2025-11-12

**Authors:** Amir Kazory, Claudio Ronco, Abhilash Koratala

**Affiliations:** 1 Division of Nephrology, Hypertension, and Renal Transplantation, University of Florida, Gainesville, FL, United States; 2 Department of Nephrology, San Bortolo Hospital and International Renal Research Institute of Vicenza (IRRIV), Vicenza, Italy; 3 Department of Medicine, University of Padova, Padova, Italy; 4 Division of Nephrology, Medical College of Wisconsin, Milwaukee, WI, United States

**Keywords:** heart failure, cardiorenal, cardionephrology, renal disease, acute kidney injury

## Introduction

The convergence of cardiovascular and renal diseases represents a common and complex challenge in contemporary medicine. Cardionephrology is the emerging subspecialty that focuses on the intersection of cardiovascular and renal diseases, addressing the bidirectional pathophysiology, clinical management, and outcomes of patients with coexisting heart and kidney disorders. With a rapidly aging population, the rising burden of diabetes and obesity, and increasing survival from acute cardiovascular events, the number of patients whose disease falls within the realm of cardionephrology has been on the rise (1, 2). Yet, in general, the educational and therapeutic frameworks of cardiology and nephrology have remained largely unchanged. Conventional training programs provide the trainees with the necessary core knowledge to provide general care for patients with renal or cardiovascular diseases. However, there is a need for focused training of interested physicians to master the specialized aspects of these exceedingly common clinical scenarios and optimize the care of patients with concomitant cardiac and renal diseases. Otherwise, they are at risk of falling through the cracks of fragmented care if they are not managed in larger academic institutions with an abundance of resources where multidisciplinary care is a possibility (3).

The time has come to redefine the field of cardiorenal medicine or cardionephrology as an integrated discipline that demands new models of research, clinical care, collaboration, and education. Herein, we provide an overview of the areas where visionary leadership and focused innovation can fundamentally reshape the landscape of this field.

## Bridging the specialty divide

While the traditional separation between cardiology and nephrology, rooted in historical and institutional structures, provides the opportunity for more specialized care, it limits our ability to deliver optimal care to patients with concomitant organ dysfunction. A new model must emerge to promote interdisciplinary collaboration at the clinical, academic, and policy levels (4). If resources allow, integrated cardiorenal clinics, co-managed care pathways, and multidisciplinary rounds should become the norm, rather than the exception. Furthermore, shared data systems and cross-specialty quality metrics are essential to fostering efficient collaboration. Not only will this integrated strategy enhance patient care, but it will also drive the next wave of research and innovation.

## Redefining cardiorenal syndrome

Cardiorenal syndrome (CRS) is at the core of cardiorenal medicine. Current classifications of CRS, based on the directionality and timing of organ dysfunction, offer a helpful framework to capture the full spectrum of cardiorenal interactions. Since they were first introduced almost two decades ago, there have been significant advances in the field, making it necessary to revisit these classifications (5). A number of important concepts have emerged that would potentially impact the management, outcome, or prognosis of these patients. These advances include the confounding effect of the rise in serum creatinine versus lingering congestion in the setting of acute decompensated heart failure (ADHF), the role of renal tubular injury biomarkers, as well as urinary sodium excretion and its role in guiding the therapy of patients with ADHF and prediction of diuretic resistance (6–8). As in other areas of medicine, artificial intelligence is likely to have a major impact on the research, education, and clinical care in CRS, such as machine learning-based models to predict diuretic efficiency in ADHF (9). We must move beyond static definitions to a more dynamic, pathophysiologically driven model. The future of CRS taxonomy should incorporate molecular signatures, hemodynamic profiles, and immuno-metabolic phenotypes. Only through a nuanced understanding of the mechanisms driving organ crosstalk can we develop targeted interventions that address the root causes of cardiorenal deterioration.

## Personalized risk stratification and prognostication

The integration of big data, machine learning, and multi-omics platforms is fundamentally expanding the potential for personalized medicine in patients with concomitant heart and kidney disease. Traditional risk stratification, which relies on population-based models and limited biomarkers (e.g., estimated glomerular filtration rate [eGFR] and N-terminal-pro B-type natriuretic peptide [NT-proBNP]), would be replaced by individualized risk profiles that incorporate genomics, proteomics, metabolomics, and real-time physiologic data. This approach enables the identification of unique molecular signatures and sub-phenotypes, allowing for more precise prediction of disease progression and response to therapy (10, 11).

Machine learning algorithms can synthesize data from electronic health records, wearable devices, and multi-omics assays to generate dynamic, patient-specific risk scores. For example, polygenic risk scores and urinary proteomic classifiers have demonstrated the ability to predict adverse outcomes and stratify patients by their likelihood of progression to heart failure, coronary artery disease, or end-stage kidney disease, surpassing the accuracy of conventional models (12, 13). This evolution allows for risk stratification that is not only more granular but also adaptive to changes in a patient’s clinical trajectory.

Artificial intelligence-driven decision support tools can identify patients at high risk for adverse outcomes, guide management, and inform the timing of interventions such as device implantation, transplant referral, or dialysis initiation. This personalized, data-driven approach transforms clinical care from reactive to proactive by enabling earlier detection of risk factors, more precise selection of available therapy options, and continuous monitoring for timely intervention, ultimately improving clinical outcomes and resource utilization (14, 15).

## Designing the next generation of cardiorenal trials

Clinical trials often exclude patients with significant comorbid cardiac or renal disease, leaving clinicians with limited evidence to guide treatment in this high-risk population. This is particularly true for patients with chronic kidney disease (CKD): it was recently reported that 74% of randomized controlled trials in cardiology excluded patients with CKD (16). The exclusion rate has continued to increase over the past two decades, and evidence gaps are more pronounced for those patients with advanced kidney disease and those receiving dialysis therapy (i.e., at higher risk of cardiovascular disease). The paucity of data in these patients forces clinicians to extrapolate from healthier populations. Future cardiorenal trials must embrace, rather than exclude, complexity. Trial designs should include composite endpoints relevant to both organs, such as hospitalization for heart failure and progression of CKD (17). Stratification by eGFR and cardiac function, standardized adjudication of adverse events, and inclusion of underrepresented populations will ensure that study findings will be broadly applicable and impactful.

## Therapeutic innovation across the heart-kidney axis

While the blockers of the renin-angiotensin-aldosterone system have traditionally been considered the key link between therapies aimed at the kidney and heart disease, the emergence of newer agents such as sodium-glucose transporter 2 (SGLT2) inhibitors has again highlighted the importance of targeting shared pathophysiologic pathways in cardiorenal disease, and how the salutary off-target effects could have a significant impact on the outcomes (18). This successful experience must serve as a blueprint for future therapeutic research and development. Mineralocorticoid receptor antagonists, glucagon-like peptide-1 (GLP-1) receptor agonists, and agents targeting inflammation, fibrosis, and oxidative stress represent other promising avenues (19–21). Novel drug delivery systems and gene editing technologies may accelerate the pipeline from bench to bedside. Ultimately, our therapeutic armamentarium must reflect the complexity and interdependence of the heart-kidney axis (22, 23).

## Addressing disparities in access to cardiorenal therapies

While there is a disproportionate burden of cardiorenal disease in specific populations, disparities also persist in access to specialized care and novel therapies (24). Addressing social determinants of health, such as socioeconomic status, remains imperative for achieving equitable health outcomes (25). Health equity initiatives should be customized to local resources and the specific needs of the populations, and could include community-based screening programs, mobile cardiorenal units, education, and expanded telehealth services that have seen increased use following the recent pandemic (26). Equitable access to advanced therapies, from novel agents to transplant, would be essential to improving outcomes at a population level.

## Advancing heart-kidney transplantation strategies

Combined and sequential heart-kidney transplantation continues to evolve, with recent consensus statements and guidelines emphasizing the importance of standardized, evidence-based criteria for candidate selection and organ allocation to ensure equitable access and optimal outcomes (27–29). The United Network for Organ Sharing (UNOS) has formalized eligibility criteria for simultaneous heart-kidney transplantation (SHKT), primarily based on the severity and chronicity of kidney dysfunction, with eGFR thresholds and evidence of intrinsic kidney disease guiding listing decisions (30). Multidisciplinary evaluation by cardiology and nephrology teams is essential to distinguish reversible cardiorenal syndrome from irreversible kidney injury, and to provide nuanced counseling regarding risks, including dialysis-requiring kidney failure after heart transplantation and the potential for subsequent kidney transplantation under the Safety Net policy (31).

Efforts to expand the donor pool include the adoption of advanced organ preservation techniques and ongoing research into xenotransplantation, which has shown promise in overcoming immunological barriers through genetic engineering and novel immunosuppressive protocols (32). Artificial intelligence and biomarkers are emerging as tools to refine patient selection, predict outcomes, and personalize post-transplant care (33). Ultimately, optimizing heart-kidney transplantation strategies would need coordinated input from cardiologists, nephrologists, transplant surgeons, and other staff, such as transplant coordinators and social workers.

## Leveraging advanced imaging and functional assessment

Non-invasive assessment of cardiorenal patients relies on advanced imaging and biomarker techniques to evaluate hemodynamics, perfusion, and fibrosis. Cardiac magnetic resonance imaging is useful for quantifying ventricular function and myocardial fibrosis, with non-contrast T1 mapping enabling safe evaluation in advanced kidney disease (34). Contrast-enhanced imaging modalities, such as PET/CT and multiparametric magnetic resonance imaging, allow simultaneous non-invasive assessment of myocardial and renal perfusion, tissue oxygenation, and fibrosis, supporting early detection and monitoring (35–37).

In parallel, multi-organ point-of-care ultrasound (POCUS) is emerging as a practical bedside tool for evaluating congestion (38, 39). Focused cardiac ultrasound allows rapid assessment of ventricular function and filling pressures, lung ultrasound detects pulmonary congestion by quantifying B-lines and identifying pleural effusions, and venous Doppler of the hepatic, portal, and intrarenal veins (collectively referred to as venous excess ultrasound or VExUS) quantifies systemic venous congestion that contributes to kidney injury. Renal resistive indices may also provide information on renal perfusion, although they are nonspecific and must be interpreted in context. POCUS often reveals congestion, including subclinical changes, before it is apparent on physical examination or conventional imaging such as chest radiography (40, 41). This provides a more accurate real-time hemodynamic profile that can then guide decongestive strategies, tailor ultrafiltration, and monitor therapeutic response. Functional biomarkers, including serum and urinary markers of tubular injury, complement imaging by providing molecular insights into disease activity (34). Moreover, artificial intelligence is increasingly used to automate image analysis, improve diagnostic accuracy, and enable earlier detection of subclinical dysfunction. The American Heart Association highlights the integration of these modalities as essential for comprehensive management and risk stratification in cardiorenal syndrome (42). Future studies should focus on improving hybrid imaging modalities and portable devices for point-of-care, real-time monitoring, which may further enhance risk stratification and guide therapy.

## Investigating environmental and lifestyle determinants

While the importance of a healthy diet in preventing cardiovascular disease has long been recognized, accumulating data points to environmental toxins, physical activity, and psychosocial stress as important determinants of health affecting both cardiovascular and renal outcomes. The escalating prevalence of CKD of unknown origin (CKDu) in agricultural communities, characterized by exposure to heat stress, dehydration, agrochemicals, heavy metals, and contaminated water, highlights the need for robust environmental studies within cardiorenal research frameworks (43, 44). Epidemiological studies have revealed that occupational and environmental exposures, such as repeated heat and dehydration, pesticide use, and heavy metal contamination, are associated with the development and progression of CKD, particularly in populations engaged in manual labor under adverse conditions (44). To address these complex multifactorial risks, precision lifestyle interventions are essential. The integration of digital health platforms, wearable technologies, and behavioral economics strategies offers promising avenues to empower patients, facilitate sustained behavioral changes, and mitigate modifiable risk factors such as poor diet, physical inactivity, and tobacco use (45, 46). In line with this concept, the American Heart Association advocates for technology-enabled interventions to promote heart-healthy behaviors and reduce disparities in cardiovascular health (45). Furthermore, upstream interventions targeting social determinants of health, such as access to safe water, improved working conditions, and reduction of environmental exposures, are essential for reducing the downstream burden of cardiorenal disease (47).

## Training the next generation of cardiorenal leaders

The future of cardionephrology depends on cultivating a workforce that has expertise in the research and clinical care of the heart and the kidney. Fellowship programs, continuing medical education, and medical school curricula must evolve to reflect the integrated nature of cardiorenal interactions. This requires not only updating didactic content but also embedding clinical rotations and case-based learning that emphasize the bidirectional pathophysiology and management strategies unique to cardiorenal medicine. Interdisciplinary mentorship, joint conferences, and collaborative research initiatives are likely to help nurture the next generation of leaders (3). It also requires the new workforce be familiar with the emerging concepts and more specialized innovative strategies such as extracorporeal therapies related to hemoadsorption and miniaturized devices dedicated to ultrafiltration therapy (48, 49). Developing dedicated cardionephrology teams and cross-specialty educational tracks is essential for fostering the early identification and joint management of patients with cardiorenal disease (42). Cross-training among nephrology and cardiology trainees and allied health professionals would ensure a pipeline of skilled clinicians equipped to address the complex needs of this patient population (4). Moreover, the development of national quality benchmarks and the implementation of interdisciplinary care models will be critical for improving outcomes and reducing the burden of cardiorenal disease.

## Conclusion

Cardionephrology is no longer a niche or secondary specialty; it is a defining challenge of 21st-century healthcare. To meet this challenge, we must revisit our processes, rethink our science, and deepen our commitment to both innovation and equity. By addressing the key issues outlined above, we can move toward a future where research is more inclusive, care is more integrated, and outcomes are more equitable. The path forward requires qualities such as vision, collaboration, and leadership that we must cultivate to unlock the full potential of cardionephrology and transform the lives of millions.
